# Decoding Alzheimer's Disease With Depression: Molecular Insights and Therapeutic Target

**DOI:** 10.1111/jcmm.70454

**Published:** 2025-03-12

**Authors:** Zekun Li, Hongmin Guo, Yihao Ge, Xiaohan Li, Fang Dong, Feng Zhang

**Affiliations:** ^1^ Department of Rehabilitation Medicine The Third Hospital of Hebei Medical University Shijiazhuang P. R. China; ^2^ Department of Clinical Laboratory Medicine The Third Hospital of Hebei Medical University Shijiazhuang P. R. China

**Keywords:** Alzheimer's disease, depression, immune cell infiltration analysis, machine learning algorithms, mendelian randomization, predictive biomarkers

## Abstract

The purpose of this study was to recognise predictive biomarkers and explore the promising therapeutic targets of AD with depression. We confirmed a positive correlation between AD and depression through MR Analysis. Through WGCNA analysis, we identified 1569 genes containing two modules, which were most related to AD. In addition, 1629 depressive DEGs were also identified. In these genes, 84 genes were shared by both AD and depression, which were screened by the Degree algorithm, MCC algorithm, and four machine learning algorithms. Two genes (ITGB5 and SPCS1) were confirmed as predictive biomarkers with AUC > 0.7. Furthermore, the nomogram indicated that ITGB5 and SPCS1 are good biomarkers in diagnosing AD with depression. Four drugs targeted at ITGB5 were determined by the DGIdb website. In conclusion, we identified two predictive biomarkers for AD with depression, thus providing promising therapeutic targets for AD with depression.

## 1 Introduction

The main characteristic of depression is a persistently low mood that persists for a long period of time, accompanied by decreased interest and loss of pleasure, often affecting the individual's work, school and social functioning. There are three grades of severity: mild, moderate, and severe. This study focused on major depressive disorder (MDD). MDD is a highly prevalent mental illness characterised by a combination of emotional, anxiety, cognitive, sleep, and appetite symptoms lasting for more than 2 weeks [1]. MDD is considered a leading contributor to global disability, impacting around 350 million individuals and resulting in more than 800,000 suicides annually [2, 3]. Additionally, MDD severely impairs function in patients and reduces quality of life, with a higher relapse rate compared to any other disease [4, 5].

AD is a classic neurodegenerative disorder with a strong genetic risk of about 60%–80% [6]. AD is the main contributor to dementia, which is responsible for 50%–75% of dementia cases [7]. As a common neurodegenerative disorder, AD poses a significant and escalating public health challenge worldwide, resulting in substantial consequences for both individuals and communities [8]. Recent data has shown that the occurrence of dementia is projected to increase twofold in Europe and triple globally by the year 2050 [7]. The hallmark biological indicators of AD are neurofibrillary tau pathology and deposition of beta amyloid [9]. While there have been notable strides in biomarkers and amyloid imaging for AD [10, 11, 12, 13], there still remains a lack of definitive diagnostic tests or biological markers for AD. The lifelong diagnosis of AD is based on clinical examination, causing great delays in identifying the condition and leading to unavoidable losses on both individual and societal levels [14, 15].

As we all know, the diagnosis of depression mainly depends on the depression scale. However, the depression scale has been questioned to some extent, and the key problem lies in the subjectivity of diagnosis. Depression is a heterogeneous disorder with different aetiologies and pathogenesis, and the symptoms and severity vary greatly in different stages and different people. When the heterogeneity of depression is ignored, there are great limitations and errors in the diagnostic results [16, 17]. MDD and AD are common in the elderly and often occur together [18, 19], with about a quarter of people with AD who are also diagnosed with MDD [20]. Unfortunately, because AD with depression still lacks specific diagnostic criteria and treatment guidelines, the diagnosis and management of AD with depression are difficult. Therefore, the discovery of sensitive and specific predictive biomarkers for AD with depression is urgently required.

Mendelian randomization is a statistical method that utilises genetic variation as an instrumental variable to infer causality between exposure factors and outcomes [21]. Firstly, this study used MR analysis to investigate the causal relationship between AD and depression. It is well known that bioinformatics approaches provide powerful tools for understanding the intricate molecular networks and common pathophysiological mechanisms involved in complex diseases [22]. By combining various omics data, such as genomics, transcriptomics, proteomics and metabolomics, bioinformatics analyses can offer comprehensive insights into the causes, development, and treatment outcomes of diseases. In this study, we utilised a variety of bioinformatics methods and machine learning algorithms to explore the co‐occurrence of AD and depression with the goal of identifying potential early predictive biomarkers for AD in depression patients and understanding the immune mechanisms involved. The findings of this study can potentially facilitate the discovery of diagnostic markers for AD in depression.

## 2 Methods

### 2.1 Mendelian Randomisation (MR)

To examine the causal relationship between AD and depression, we performed MR analysis utilising the TwoSampleMR software package. Firstly, data on exposure variables were gained from the GWAS database with the aim of identifying SNPs related to exposure factors. The data related to the outcome variables were then gained from the GWAS database, and the presence of the relevant SNPs was identified. The detailed information of the datasets was shown in Table S1. Finally, eligible SNPs were screened and multitudinous statistical means were utilised to synthetically determine the causal relationship between AD and depression. The inverse variance weighted (IVW) method was used to generate the primary results. The MR Egger, weighted median, etc. were used for the supplementary results.

### 2.2 Microarray Data

Four public microarray datasets (GSE132903, GSE98793, GSE1297 and GSE122063) were retrieved from the NCBI Gene Expression Omnibus (GEO) database. They include three AD datasets and one depression dataset. The details of these datasets are shown in Table 1.

**TABLE 1:** Microarray information.

GEO ID	Platform	Participants	Tissues	Type
GSE132903 (test dataset)	GPL10558	97 AD and 98 ND	Brain	mRNA
GSE98793	GPL570	128 depression and 64 control	Blood	mRNA
GSE1297 (validation dataset)	GPL96	22 AD and 9 control	Brain	mRNA
GSE122063 (validation dataset)	GPL21827	56 AD and 44 control	Brain	mRNA

### 2.3 Weighted Gene co‐Expression Network Analysis(WGCNA)of AD

WGCNA is a biological tool for constructing gene co‐expression networks, which has been widely employed in trait and inter‐gene association analysis. In this study, the gene expression levels of 195 samples from GSE132903 were utilised as input data, with AD and non‐demented controls (ND) serving as trait data. The co‐expression network was established by the ‘WGCNA’ R package to identify AD‐related gene modules. Firstly, the hcluster function is used to perform sample clustering of outliers with the parameter ‘method = average’ to calculate the distance of outliers. Next, the optimal soft threshold is determined and the co‐expression network is constructed. The modules are then visualised by hierarchical clustering and the dynamic Treecut function. Correlation analysis is employed to identify modules related to AD. The genes associated with AD are obtained by analysing the module membership (MM) and gene significance (GS) within the module.

### 2.4 Identification of DEGs

The initial data were retrieved, and background calibration, normalisation, and log2 conversion were operated utilising the ‘affy’. Subsequently, a difference analysis on the GSE98793 dataset was carried out using the software package of ‘limma’ to distinguish the DEGs between depression and control groups. The selection criterion for DEGs was set at *p* < 0.05. Heat maps and volcano maps illustrating the expression of DEGs were generated using ‘ggplot2’ and ‘pheatmap’, respectively.

### 2.5 Functional Enrichment Analysis (EA) of Interaction Genes

The interaction genes between the AD‐related module genes identified by WGCNA and DEGs associated with depression detected by limma were obtained using Venn analysis. These interaction genes were then subjected to functional EA using GO and KEGG. Enriched GO and KEGG pathways with *p* < 0.05 were identified, and the top enriched pathways were visualised.

### 2.6 Gene Set Enrichment Analysis (GSEA)

GSEA is a functional classification method used to calculate enrichment scores of gene sets and identify different functional phenotypes. The interaction genes were divided into high expression groups and low expression groups based on the median gene expression. We performed GSEA on DEGs using the ‘GSVA’ R package and screened the top five important KEGG pathways and GO entries.

### 2.7 Construction of Protein–Protein Interaction (PPI) Network

The PPI network was generated employing the STRING database with a confidence level of 0.4. The PPI network was used to investigate the interaction between AD and depression interaction genes. Discrete genes were removed, and data was retrieved from the database and visualised using Cytoscape software. The Degree algorithm and MCC algorithm in the Cytoscape CytoHubba plugin were used to select the top 10 genes.

### 2.8 Machine Learning Algorithms Identify Candidate Predictive Biomarkers for AD With Depression

In this study, four machine learning algorithms (LASSO regression, random forest, support vector machine recursive feature elimination [SVM‐RFE], and Gaussian mixture model [GMM]) were employed to identify potential predictive biomarkers for AD with depression. The interaction genes of LASSO, random forest, SVM‐RFE and GMM are considered to be candidate biomarkers for AD with depression diagnosis.

### 2.9 Evaluation of Receiver Operating Characteristic (ROC)

The expression of candidate biomarkers in the ND group and AD group was compared, and the diagnostic value of these biomarkers was evaluated. ROC was then drawn, and the area under the ROC curve (AUC) was calculated to estimate the diagnostic value with a 95% confidence interval. To avoid bias, GSE1297 was employed to be the validation dataset. Candidate biomarkers with an AUC greater than 0.7 in both the test dataset and validation dataset were selected as predictive biomarkers.

### 2.10 Construction of Nomogram

Only predictive models with AUC greater than 0.7 in both the validation dataset and test dataset will be selected to build the nomogram using the ‘rms’. The diagnostic property of the nomogram was verified through measuring AUC.

### 2.11 Analysis of Immune Cell Infiltration

The gene set for labeling 28 immune cell types is described in supplemental Table S2. The relative infiltration abundance of immune cells in AD and ND was evaluated using the ssGSEA algorithm implemented in the GSVA R software package. This algorithm is commonly used in bioinformatics studies that investigate immune infiltration. The boxplot was used to compare the differential expression of immune cells between the two groups. To visualise the relation of different immune cells in the progression of AD, a heat map was generated using the GSVA R package. Furthermore, this study examined how diagnostic predictive biomarkers of AD with depression are related to the immune cells.

### 2.12 Drug Prediction Analysis

The interaction between drug and gene target was calculated by using the DGIdb website (https://www.dgidb.org/).

## 3 Results

### 3.1 The Causal Relationship Between AD and Depression Was Evaluated Using MR Analysis

**FIGURE 1:**
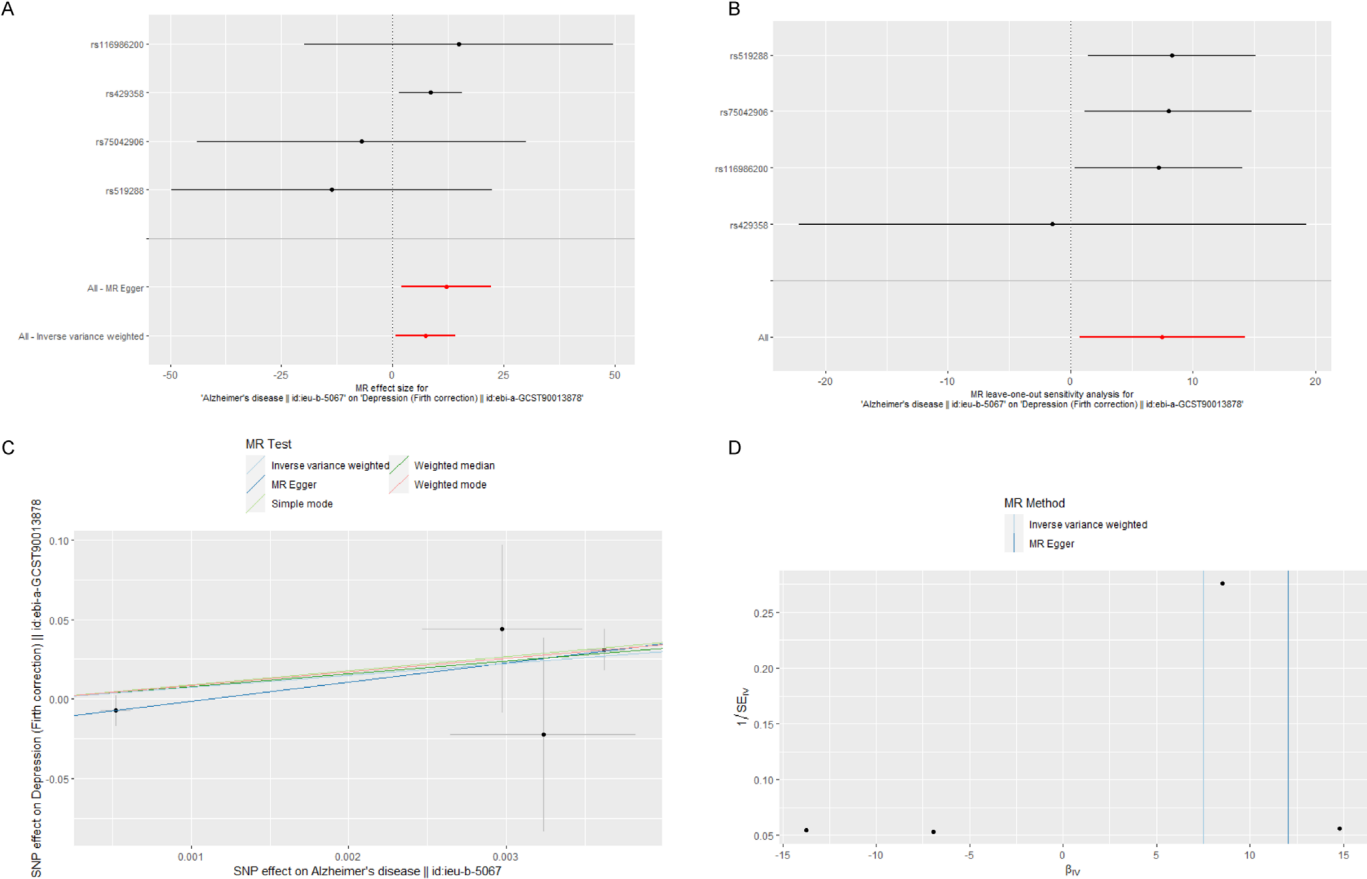
The causality of AD on depression in Europeans. (A) Forest map, the red dots represent a comprehensive estimate utilising all SNPs, utilising the IVW method, the horizontal line represents a 95% confidence interval; (B) MR leave‐one‐out sensitivity analysis for AD on depression, the black dots indicate that the IVW mean was utilised to evaluate causal influences, excluding a single specific variable in the analysis; (C) Scatter plot, the slope of different coloured lines indicates the estimation effect of different MR methods; (D) Funnel diagram; the vertical line denotes the estimated value of all SNPs.

Two‐sample MR Method was used with AD GWAS dataset (ieu‐b‐5067) as the exposure factor and depression GWAS dataset (ebi‐a‐GCST90013878) as the outcome factor. IVW results showed a positive correlation between AD and depression (Figure 1 and Table S3). To confirm this result, another depression GWAS dataset (ebi‐a‐GCST90038650) was selected as the outcome factor for MR Analysis, and the analysis results also supported a significant positive correlation between AD and depression (Table S4 and Figure S1).

### 3.2 Identification of Gene Modules Associated With AD

**FIGURE 2:**
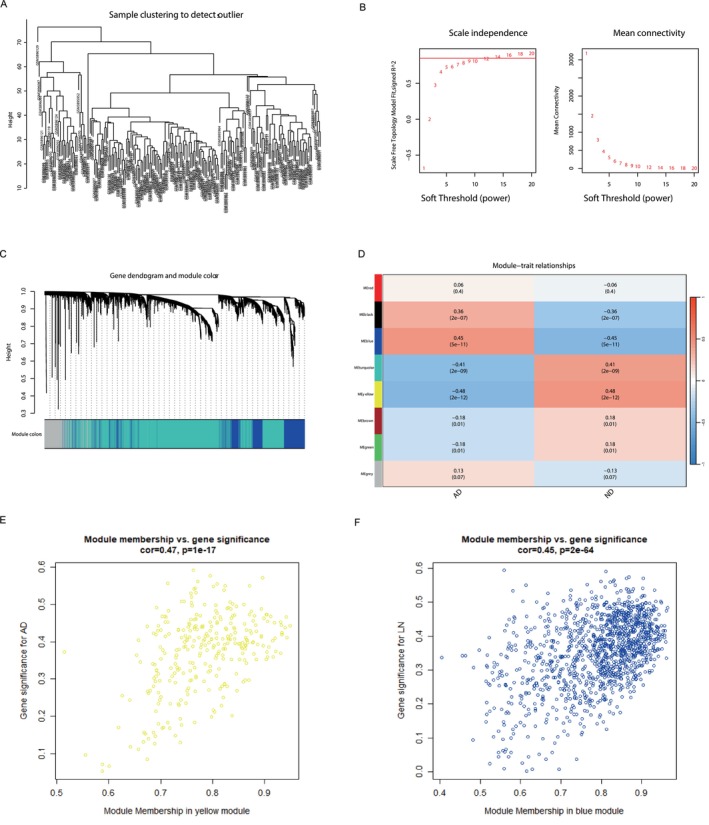
WGCNA for screening AD‐related gene modules. (A) Clustering dendrogram of the samples; (B) According to scale independence and average connectivity analysis, *β* = 14 was selected as the soft threshold; (C) Gene co‐expression modules in different colours under the gene tree; (D) The heat map described the correlation between gene modules and AD; the number in the upper bracket represented the correlation coefficient, and the number in the lower bracket showed the *p*‐value; (E) There was a negative relation between MM and AD GS; (F) There was a positive relation between MM and AD GS.

The sample clustering to detect clusters of AD and ND samples is shown in Figure 2A. No abnormal samples were detected in hierarchical clustering. The interconnected gene clusters or modules closely related to AD were recognised by WGCNA analysis. The most appropriate soft threshold power β = 14 was selected according to scale independence and mean connectivity (Figure 2B). After the modules were combined, eight AD‐related gene co‐expression modules were gained (Figure 2C,D). These modules were represented by different colours, with a significant correlation between members of the blue and yellow modules and AD, with the yellow module representing the most significant negative relation (297 genes; correlation coefficient = −0.48; *p* < 0.0001) (Figure 2E, Table S5), and the blue module representing the most significant positive relation (1272 genes; correlation coefficient = 0.45; *p* < 0.0001) (Figure 2F and Table S6).

### 3.3 Identification of Differentially Expressed Genes in Depression

**FIGURE 3:**
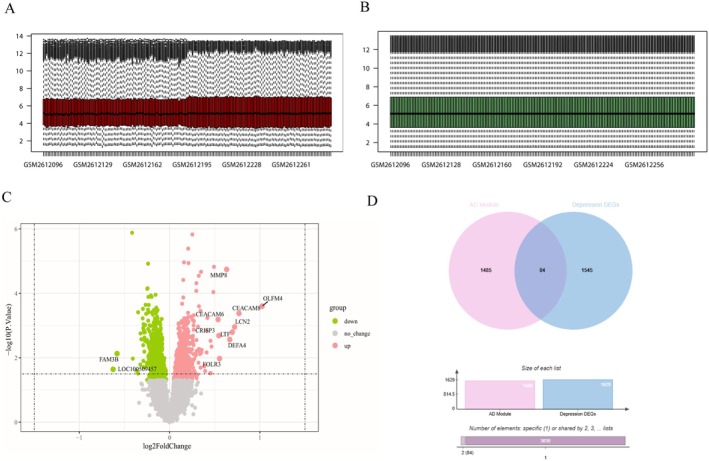
Identify DEGs between control and depression. (A) Box diagram of raw data among samples; (B) Box diagram of raw data normalised among samples; (C) The volcano map shows all the DEGs, where pink is up and green is down; (D) The interaction of AD‐related module genes identified by WGCNA and depression DEGs detected by limma was visualised using a Venn diagram.

The GSE98793 dataset was normalised and depicted as a boxplot (Figure 3A,B). We utilised limma to identify 1629 DEGs with a significance level of *p* < 0.05 between the depression and control. Out of these DEGs, 847 were activated and 782 were suppressed. The volcano map (Figure 3C) showed all the DEGs, with activated genes in red and suppressed genes in green. By intersecting the 1569 module genes associated with AD identified by WGCNA and the 451 DEGs associated with depression detected by limma, 84 interaction genes were identified (Figure 3D and Table S7).

### 3.4 Functional Enrichment Analysis of Interaction Genes

**FIGURE 4:**
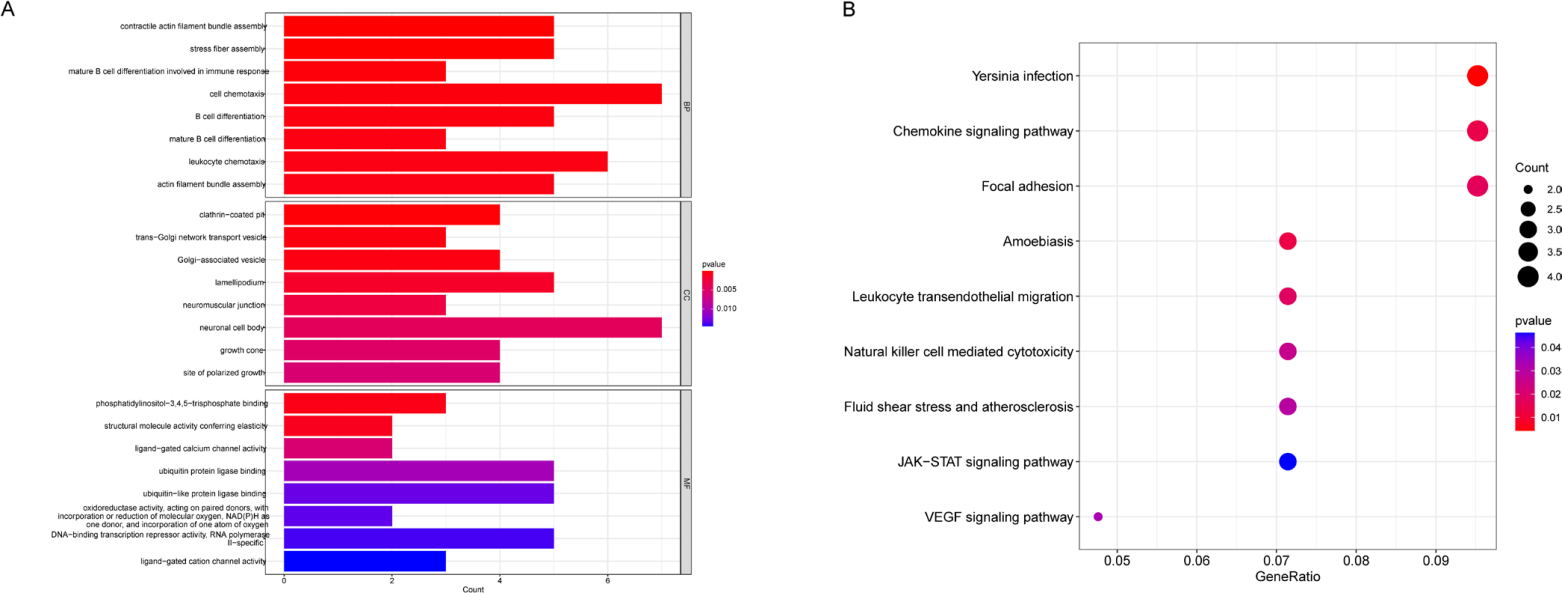
Functional enrichment analysis of 84 interaction genes. (A) GO enrichment analysis, the top 8 enrichment classes (biological processes, cell components, and molecular functions) are visualised. The *x*‐axis represents the count and the *y*‐axis represents the different ontologies. The size of the circle represents the count of genes, and the colour represents the *p*‐value. (B) KEGG analysis of interaction genes. The *x*‐axis represents the gene ratio and the *y*‐axis represents the different ontologies.

Functional enrichment analysis of 84 interaction genes was conducted with GO and KEGG. GO enrichment analysis demonstrated that the interaction genes were primarily enriched in a few items: (1) biological processes, cell chemotaxis, leukocyte chemotaxis, B cell differentiation, etc. (2) cellular components, neuronal cell body, lamellipodium, Golgi−associated vesicle, etc., and (3) molecular function, ubiquitin protein ligase binding, etc. (Figure 4A). KEGG signalling pathways included Chemokine signalling pathway, Focal adhesion, Leukocyte transendothelial migration, Natural killer cell mediated cytotoxicity, Fluid shear stress and atherosclerosis, JAK–STAT signalling pathway, VEGF signalling pathway, etc. (Figure 4B).

### 3.5 GSEA

**FIGURE 5:**
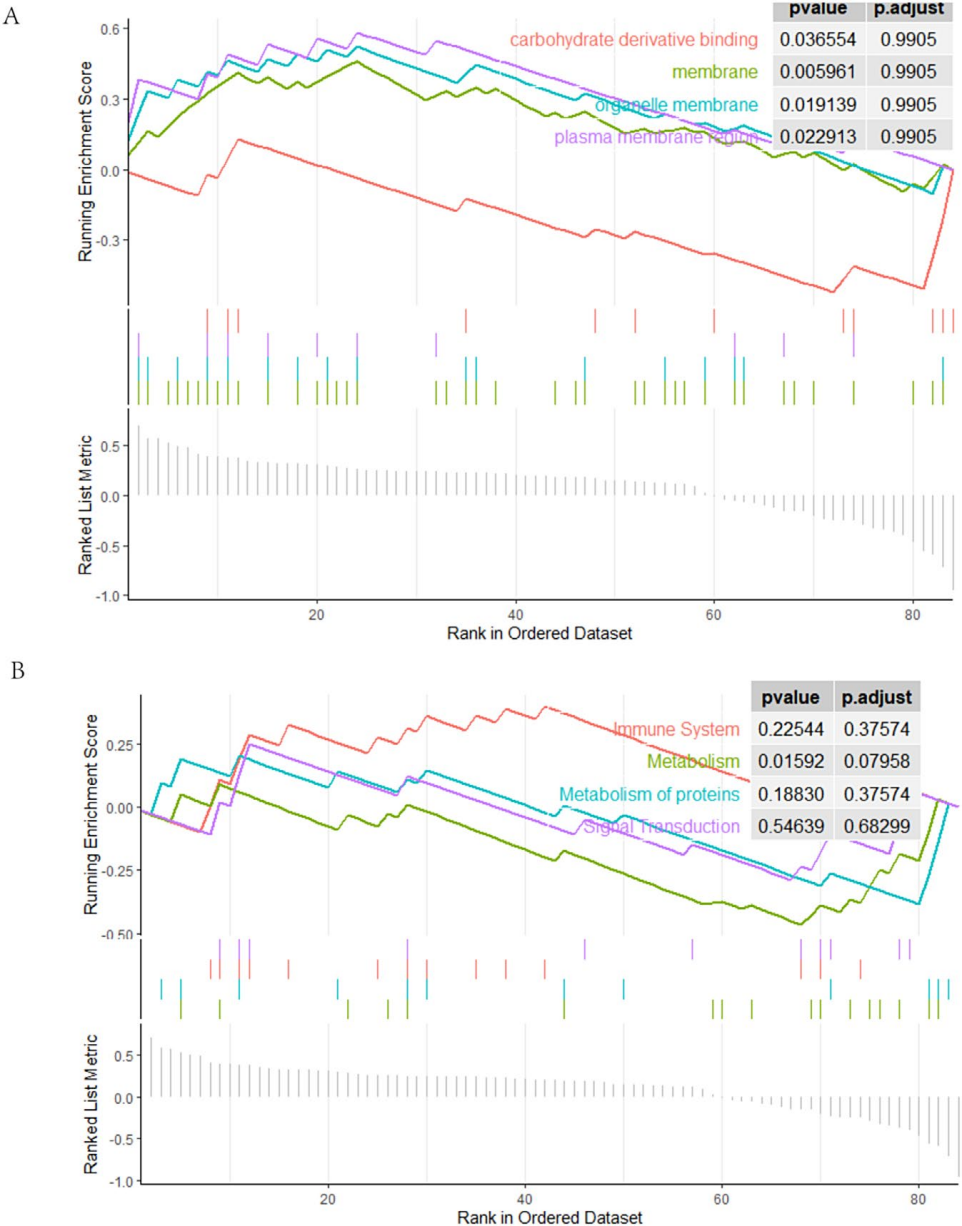
GSEA analysis. (A) GO gene set enrichment analysis; (B) Rectome gene set enrichment analysis.

We further investigated the most significant enrichment functions among 84 interaction genes by GSEA. In combination with the main biological functions and *p*‐values of the pathway, we visualised the top four terms, including carbohydrate derivative binding, membrane, organelle membrane, and plasma membrane region (Figure 5A). In addition, GSEA of Rectome was performed, and the top four items were immune system, metabolism, metabolism of protein, and signal transduction (Figure 5B).

### 3.6 Construction of PPI Network

**FIGURE 6:**
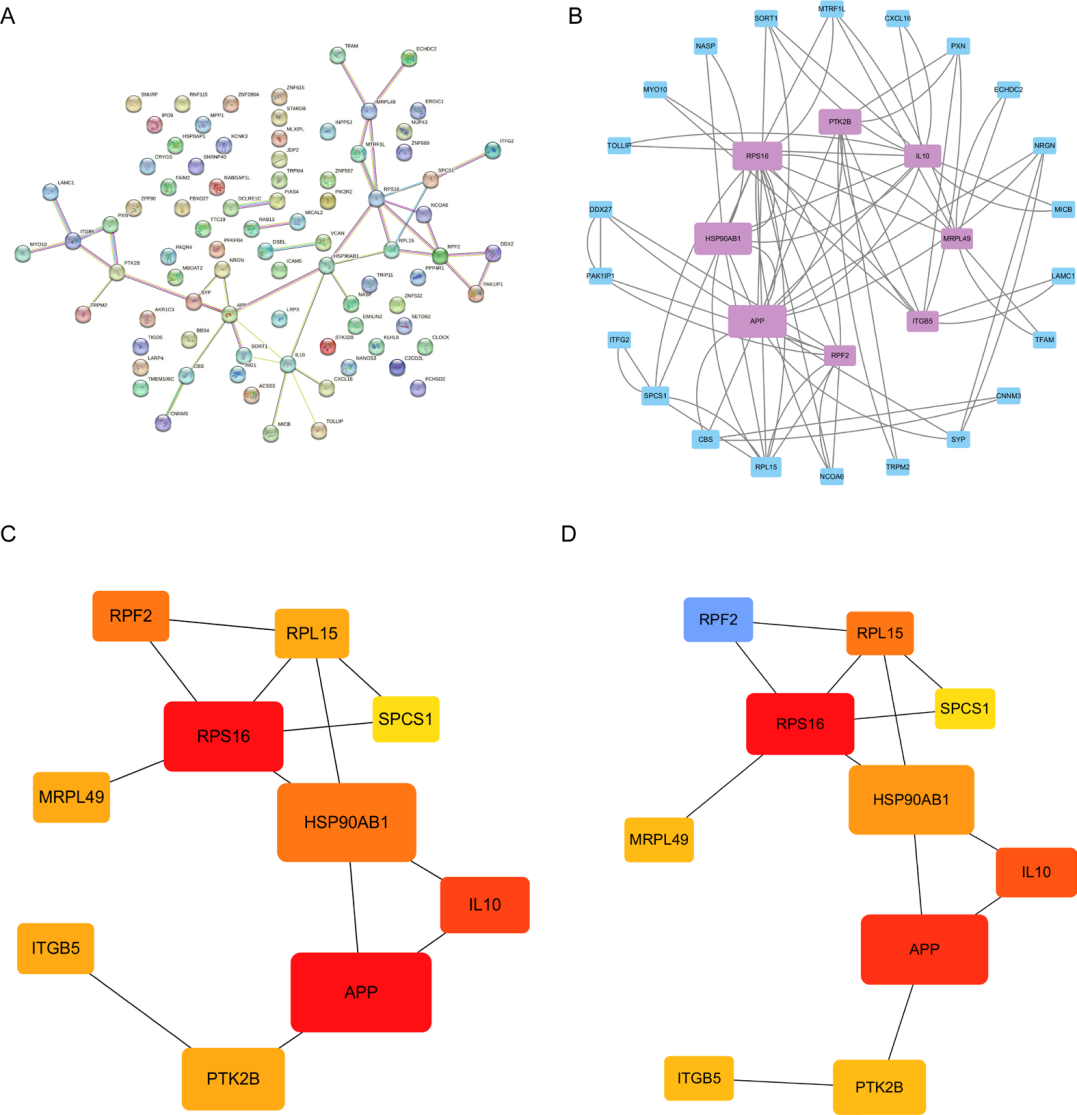
Construction of protein–protein interaction (PPI) network diagram. (A) PPI network of 84 interaction genes; (B) The PPI network was constructed and visualised using Cytoscape software (The bigger the rectangle, the higher the Betweenness Centrality score); (C) Degree algorithm results. (D) MCC algorithm results.

A PPI network with 84 nodes and 42 edges was constructed (Figure 6A). Fifty‐four of the 84 genes were unrelated to other molecules and did not form molecular networks. And 30 proteins were identified after the isolated proteins were removed from the PPI network (Figure 6B). The Cytoscape CytoHubba plug‐in was used to identify hub genes. The top 10 genes with the highest scores (Degree algorithm and MCC algorithm) were selected as hub genes (Figure 6C,D).

### 3.7 Identification of Candidate Biomarkers via Machine Learning

**FIGURE 7:**
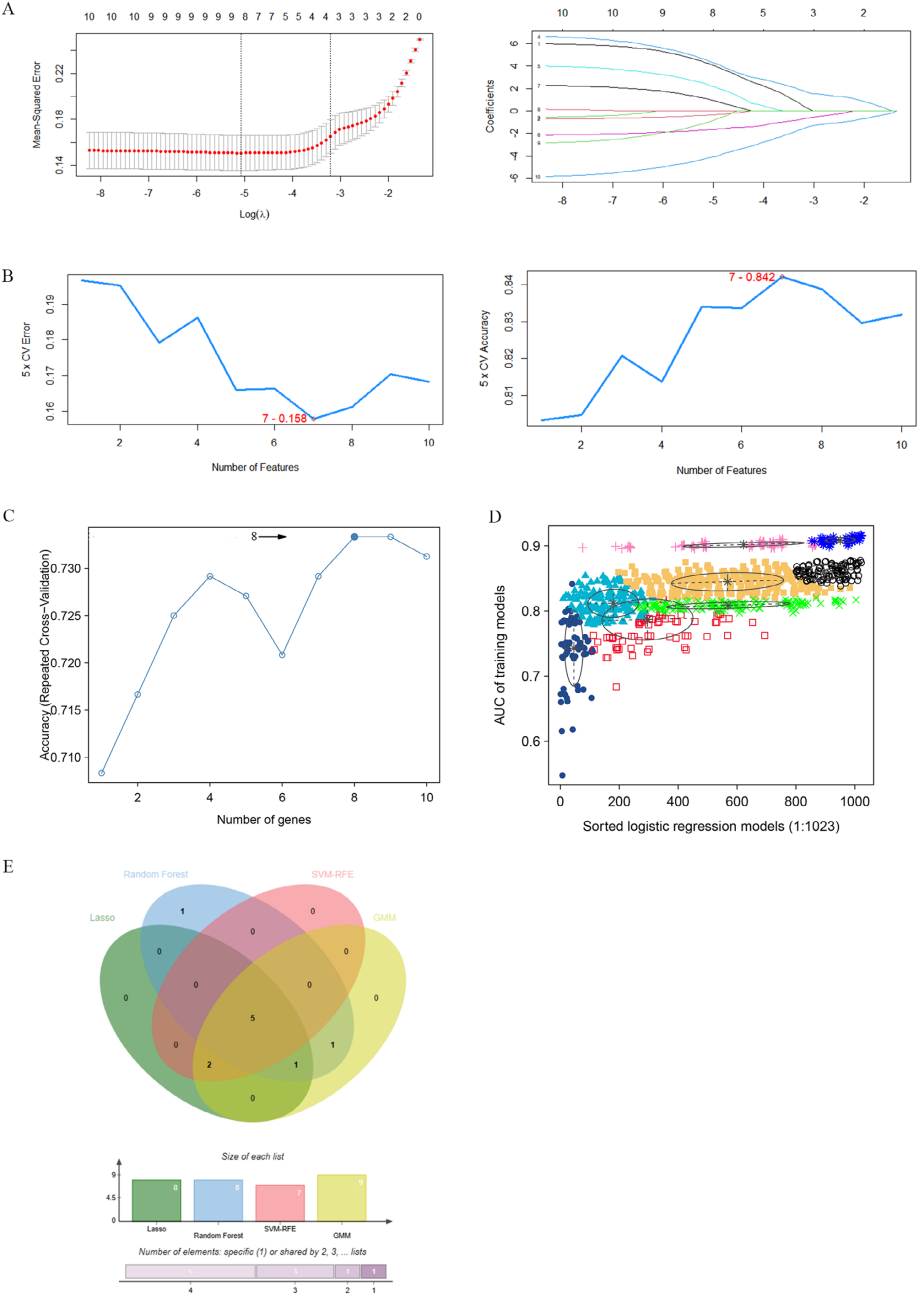
Candidate biomarkers for AD with depression were selected by machine learning method. (A) The biomarkers were screened by LASSO regression analysis according to the top 10 node genes. The number of genes (*n* = 8) corresponding to the lowest binomial deviation was the most suitable for the diagnosis of AD with depression; (B) Through the SVM‐RFE algorithm, the number of genes (*n* = 7) with minimised error and maximised accuracy following 100 folds was regarded as the most appropriate candidate for diagnosing AD with depression; (C) The random forest algorithm; (D) GMM classifier determined that the average accuracy of a single characteristic gene among the eight combinations; (E) Venn plot displayed that five candidate biomarkers were identified through four algorithms.

Eight genes were recognised through LASSO regression analysis (Figure 7A), and seven genes were recognised by SVM‐RFE means with the minimised error and maximised accuracy following 100 iterations to diagnose AD with depression (Figure 7B). Using the random forest method, gene importance scores were calculated and 8 candidate genes were selected by ranking the genes according to their importance (Figure 7C). The Gaussian mixture model (GMM) classifier identified a single feature gene set in the eight combinations with an average accuracy of 0.9162634 (Figure 7D). Finally, the five interaction genes were screened by the four machine learning algorithms, namely integrin subunit beta 5 (ITGB5), signal peptidase complex subunit 1 (SPCS1), protein tyrosine kinase 2β (PTK2B), ribosomal protein S16 (RPS16) and ribosome production factor 2 homologue (RPF2), which were regarded as candidate biomarkers for AD with depression (Figure 7E and Table S8).

### 3.8 Assessment of the Diagnostic Value of Biomarkers

**FIGURE 8:**
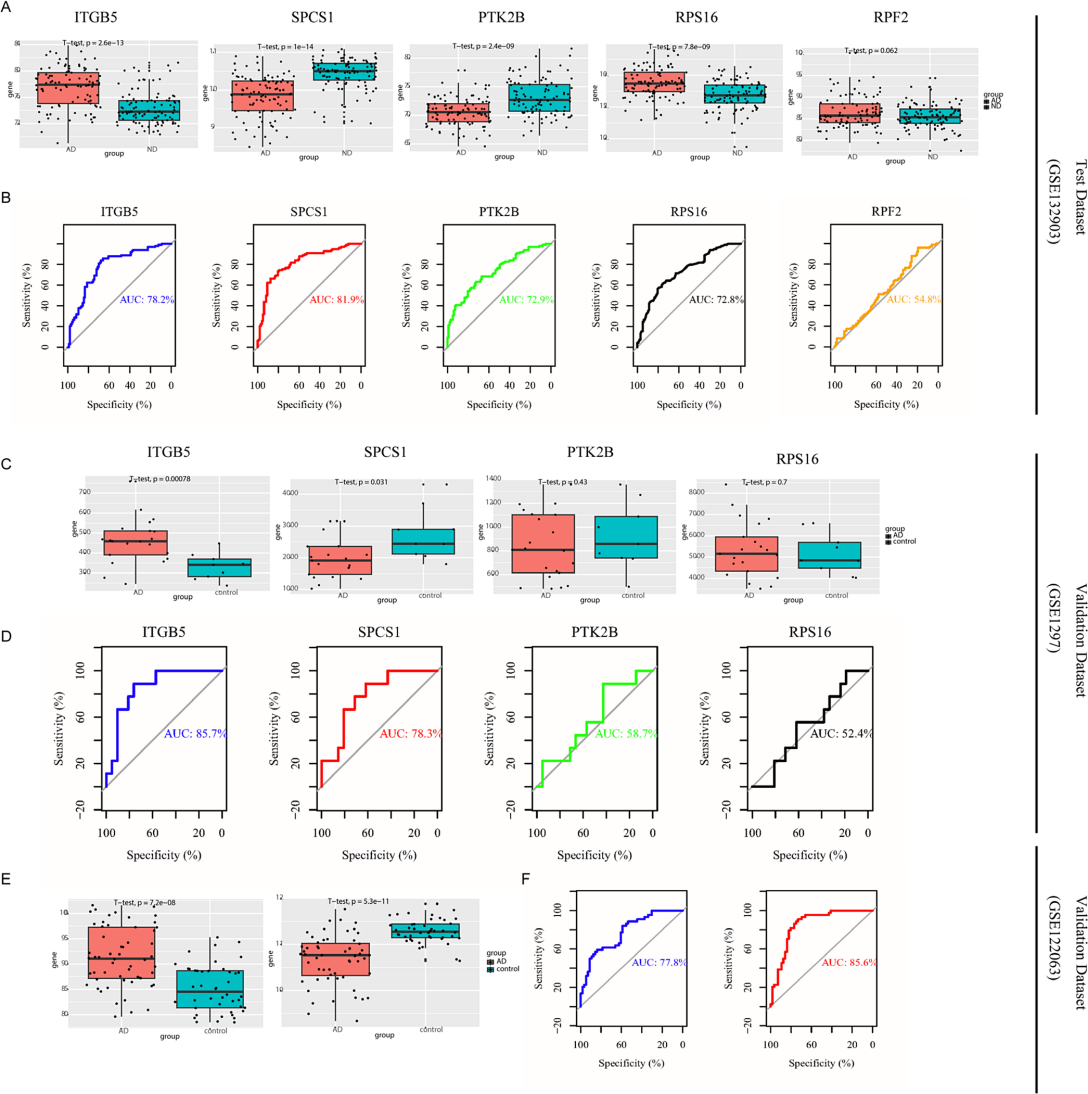
The diagnostic value of candidate biomarkers for AD. (A) The levels of five candidate biomarkers in the GSE132903; (B) ROC curves for 5 predictive biomarkers in the GSE132903; (C) The levels of four biomarkers in the GSE1297; (D) ROC curves for four biomarkers in the GSE1297; (E) The levels of biomarkers (ITGB5 and SPCS1) in the GSE122063; (F) ROC curves for biomarkers (ITGB5 and SPCS1) in the GSE122063.

The expression levels of five candidate biomarkers in the AD and ND groups were compared in the GSE132903 test dataset. Except for RPF2, the expression of the remaining predictive biomarkers showed statistically significant differences between the two groups (Figure 8A). The ROC curve was then generated and the AUC was evaluated to assess the diagnostic valuation of the five candidate biomarkers. AUC > 0.7 indicates good diagnostic value (Figure 8B). Then, the expression of four genes other than RPF2 was assessed in GSE1297, and no differences in the expression of RPS16 and PTK2B were found between the AD and control groups (Figure 8C). These two biomarkers (RPS16 and PTK2B) have an AUC of less than 0.7, while the remaining two biomarkers (ITGB5 and SPCS1) have a slightly higher AUC (Figure 8D). The expression of ITGB5 and SPCS1 was evaluated in GSE122063, and there is a significant difference in the level of these two biomarkers between the AD and control group (Figure 8E). Moreover, the AUC of ITGB5 and SPCS1 is greater than 0.7, which has good diagnostic value (Figure 8F). Moreover, the expression trend of these two biomarkers in depression is consistent with that in AD. Therefore, ITGB5 and SPCS1 were used as predictive biomarkers of AD with depression (Figure S2).

### 3.9 Construct a Nomogram to Assess the Diagnostic Value

**FIGURE 9:**
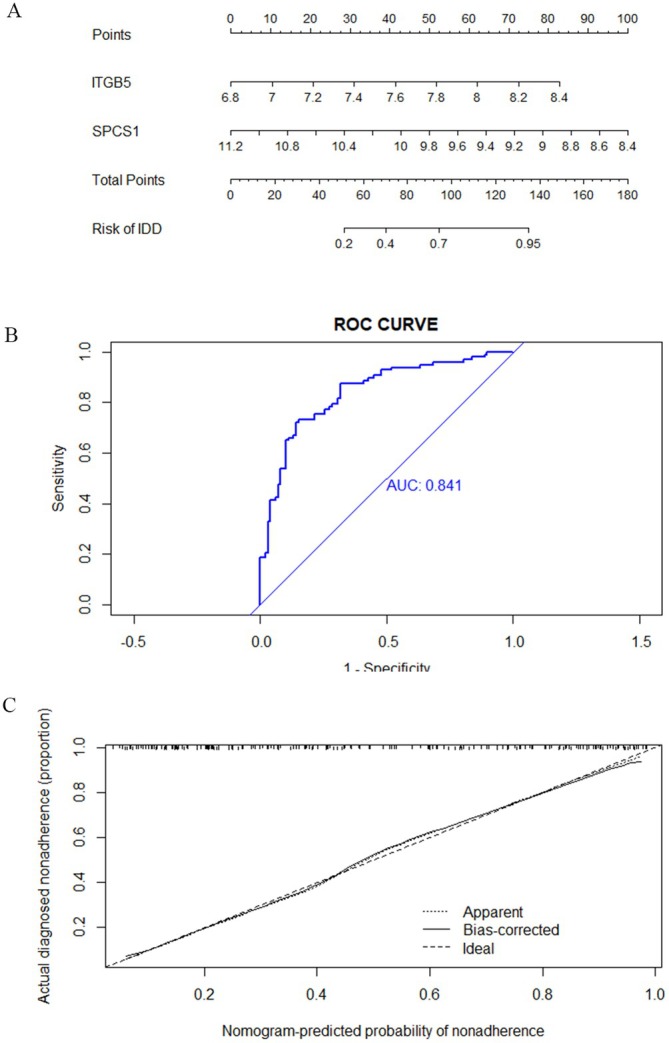
Construction of nomogram and evaluation of diagnostic performance. (A) After several rounds of screening, the nomogram was built according to the two predictive biomarkers; (B) The ROC curve of the nomogram.

After several rounds of screening, these two genes (ITGB5 and SPCS1) were finally selected to build a nomogram. The expression of each gene corresponds to the score in the nomogram (Figure 9A). The AUC of the nomogram was 0.841, indicating that ITGB5 and SPCS1 are good biomarkers in diagnosing AD with depression (Figure 9B). The correction curve showed a small error between the exact and the estimated risk for AD with depression, showing that the nomogram model was more accurate in forecasting AD with depression (Figure 9C).

### 3.10 Immune Cell Infiltration Analysis

**FIGURE 10:**
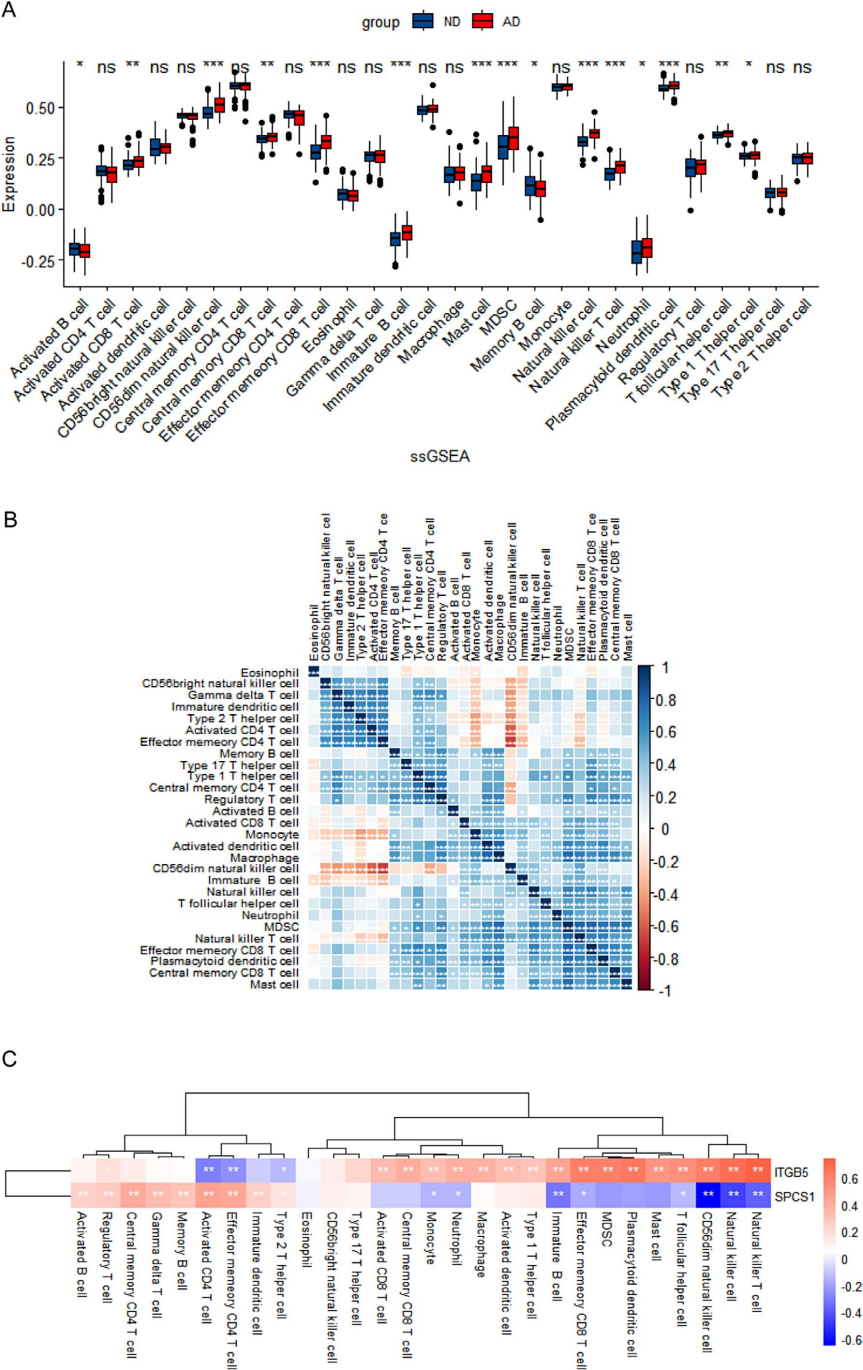
Immune cell infiltration analysis between AD and ND. (A) Comparison of the proportion of 28 kinds of immune cells between the AD group and the ND group; (B) The correlation of 28 kinds of immune cells in AD is shown by heatmap; (C) Heat map of correlation between 2 predictive biomarkers and 28 types of immune cells.

According to the results of enrichment analysis, depression‐associated genes could modulate the pathogenesis of AD, which were mainly enriched in immune regulation. Therefore, the immune cell infiltration analysis was conducted to further illustrate the immune modulation of AD. The level of plasmacytoid dendritic cells T follicular helper cells, and other immune cells was higher in AD group than ND group, and the level of activated B cells and memory B cell was lower in AD group than ND group (Figure 10A). Subsequently, correlations between different immune cells in AD were evaluated (Figure 10B). In summary, there are differential infiltration levels of various immune cells in AD patients, which can be used to investigate promising AD treatments. In addition, the expression of the 2 predictive biomarkers might influence the level of AD‐infiltration immune cell types (Figure 10C). ITGB5 was significantly correlated with 20 immune cells, among which Natural killer T cells had the strongest correlation (*r* = 0.7455, *p* < 0.01). In addition to Activated CD4 T cells, Effector memory CD4 T cells, and Type 2 T helper cells, most of which were positively correlated (3/20). SPCS1 was positively correlated with 9 kinds of immune cells and negatively correlated with eight kinds of immune cells. The strongest correlation was found in CD56dim natural killer cells (*r* = −0.6522, *p* < 0.01).

### 3.11 Potential Drug Screening for the Key Genes

The DGIdb website was used to predict potentially effective therapeutic agents for ITGB5 and SPCS1. There are four potential therapeutic agents for ITGB5, including CILENGITIDE, INTETUMUMAB, ABITUZUMAB and INTEGRIN RECEPTOR ANTAGONIST GLPG0187. No potential drugs for SPCS1 were retrieved (Table 2).

**TABLE 2:** Drugs information.

Key Gene	Drug	Type
ITGB5	Cilengitide	Inhibitor
ITGB5	Abituzumab	Inhibitor
ITGB5	D‐Tyrosine	Inhibitor
ITGB5	Intetumumab	Inhibitor

## 4 Discussion

It is well known that depression often accompanies the development of AD [23, 24, 25]. In this study, we investigated the causal relationship between AD and depression using MR. The results of the MR analysis showed a significant positive correlation between AD and depression. The patients suffering from AD with depression present a substantial challenge in clinic. The potential mechanisms remain unclear, and the resultant interventions are imperative. This study aims to offer novel insights into the biomarkers and therapeutic targets of AD with depression through bioinformatics analysis. We utilised bioinformatic analysis and machine learning methods to screen key targets and construct ROC curves and a nomogram to evaluate the diagnostic value of biomarkers. The most notable finding is that we identified two predictive genes (ITGB5 and SPCS1) and formed a nomogram for diagnosing AD with depression. In addition, we used the Genecards database, which contains many drugs, to explore therapeutic drugs targeted at identified genes.

ITGB5 is a member of the integrin family, which are cell surface receptors that mediate cell‐extracellular matrix and cell–cell adhesion [26]. Specifically, ITGB5 forms heterodimers with integrin alpha subunits to create functional integrin receptors, playing crucial roles in various cellular processes such as cell migration, proliferation, differentiation, and immune response regulation [27, 28]. ITGB5 has been implicated in numerous physiological and pathological processes, including embryonic development, wound healing, cancer metastasis, and inflammation [29, 30, 31]. Therefore, ITGB5 dysregulation is associated with a variety of diseases, making ITGB5 an important target for therapeutic intervention. To date, there is limited direct evidence linking ITGB5 to AD with depression. However, given the emerging understanding of the role of cell adhesion molecules and extracellular matrix interactions in neurodegenerative disorders and mood disorders [32, 33, 34], ITGB5, as a cell surface receptor involved in these processes, may potentially contribute to the pathogenesis of AD with depression. ITGB5 plays a role in neuroinflammation by modulating the activation of glial cells, such as microglia [35], which are implicated in both AD and depression. In AD, microglial activation contributes to amyloid‐beta deposition and neurodegeneration [36], and microglia can sense depression‐related stressors and induce immune responses, resulting in depression [37, 38]. In depression individuals, microglia may become activated, releasing pro‐inflammatory cytokines and other molecules that can affect mood‐regulating circuits in the brain [39]. Furthermore, overactivation of microglia disrupts neuroplasticity and synaptic remodelling [40, 41], leading to depression and cognitive impairment. Therefore, ITGB5 may be a promising therapeutic target for AD with depression. Therefore, the molecular mechanisms underlying ITGB5's dysregulation in AD with depression is worthy of further investigation, which could provide valuable insights into new therapeutic targets for complex neurodegenerative and psychiatric disorders.

SPCS1 (Signal Peptide Peptidase Like 1) is a signal peptide peptidase‐like protein belonging to the membrane protein family, actively involved in intracellular protein processing pathways [42, 43]. The primary function is to cleave the signal peptides within the endoplasmic reticulum, facilitating the accurate localisation of newly synthesised proteins to various cellular compartments. Additionally, SPCS1 is implicated in intracellular signalling transduction and protein degradation processes [44]. In the pathogenesis of AD and depression, mounting evidence suggests an intimate association between aberrations in intracellular protein processing pathways and disease progression [45, 46]. In AD, the accumulation of misfolded proteins like β‐amyloid and tau can trigger ER stress and exacerbate neurodegeneration [47], while ER dysfunction can impair neuronal function and emotional regulation in depression [48]. In this process, SPCS1 is able to help reduce the accumulation of toxic proteins, thereby offering neuroprotection [49]. Although the precise mechanisms of SPCS1's involvement in AD with depression remain incompletely understood, its significance in intracellular protein processing pathways and association with these AD with depression has garnered considerable research interest. Therefore, further investigation is necessary to elucidate the specific mechanisms through which SPCS1 impacts the development of AD with depression.

Immune regulation and inflammatory responses play key roles in the pathological mechanisms of AD with depression [50, 51, 52, 53]. Studies have shown that there is a significant inflammatory response in the brain tissue of AD mice, which is manifested by an increase in inflammatory mediators and the activation of immune cells [52, 54]. These mediators include tumour necrosis factor‐α (TNF‐α), interleukin‐1β (IL‐1β), and IL‐6, which can induce neuronal damage, promote amyloid beta aggregation, and lead to loss of synaptic function [55, 56]. According to the results of immune cell infiltration, AD patients had a higher level of natural killer T cells, neutrophils and other immune cells, and a lower level of type 1 T helper cells, activated B cells and memory B cells, which are consistent with previous studies [57, 58]. In addition, ITGB5 showed a strong correlation with several immune cell types, particularly Natural Killer T cells (*r* = 0.7455, *p* < 0.01), suggesting ITGB5 might play a role in the migration and activation of immune cells, thereby influencing the immune response in AD with depression patients. In addition, SPCS1 showed a complex relationship with immune cell infiltration in AD, being positively correlated with 9 types of immune cells and negatively correlated with 8 types of other cells, indicating the potential role of SPCS1 in maintaining immune response balance. Notably, the negative correlation between SPCS1 and CD56dim natural killer (NK) cells (*r* = −0.6522, *p* < 0.01) suggests that SPCS1 might inhibit the activation or infiltration of NK cells, thereby affecting immune responses in the brain. Because NK cells are involved in the clearance of damaged or infected cells, a reduction in NK cell activity could lead to a shift toward adaptive immunity and potentially exacerbate inflammatory conditions, thus accelerating AD with depression progression. Moreover, the role of SPCS1 in protein synthesis may impact the expression of cytokines or other inflammatory mediators, which is crucial for understanding the role of SPCS1 in the neuroinflammation of AD with depression. In summary, comprehending inflammatory signalling mechanisms could facilitate the diagnosis of AD and the development of effective therapies.

While our study provides novel insights, there are some limitations. We selected genes based on genes that were cross‐identified by four machine learning algorithms, each algorithm with its own characteristics. First, LASSO can address overfitting by selecting important variables, tending to select one and disregard the others, thus leading to an inaccurate result [59]. Second, Random Forest methods can manage high‐dimensional data and mitigate overfitting by constructing multiple decision trees through random sampling and feature selection. However, its ensemble nature often impacts its interpretability, making it challenging to discern individual decision trees [60]. Third, SVM‐RFE, a fusion of SVM and RFE, enhances interpretability by identifying crucial features while maintaining model performance. Yet, it may incur high computational costs, particularly for large datasets or datasets with numerous features, and requires careful parameter tuning [61]. Fourth, GMM offers flexibility in modelling complex data distributions, which is suitable for datasets with multiple distinct distributions. However, its computational demands can be huge, especially with a large number of parameters, and its performance may decline in the presence of overlapping data distributions. In addition, there is only two datasets used for validation. Therefore, further basic experiments and clinical trials are essential to verify the outcomes.

## 5 Conclusion

In this paper, we demonstrate a causal relationship between AD and depression through Mendelian randomization. Furthermore, we have recognised two candidate key genes (ITGB5 and SPCS1) through different bioinformatics analysis methods and machine learning algorithms. Meanwhile, we also noted that the proportion of immune cells in AD patients is abnormal. In conclusion, this study may provide promising diagnostic predictive biomarkers for patients suffering from AD with depression, which may also be a breakthrough point in exploring new treatments for AD with depression.

## Author Contributions

**Zekun Li:** conceptualization (equal), data curation (equal), formal analysis (equal), methodology (equal), software (equal), writing – original draft (equal), writing – review and editing (equal). **Hongmin Guo:** formal analysis (equal), methodology (equal), software (equal), writing – original draft (equal). **Yihao Ge:** formal analysis (equal), methodology (equal), software (equal), writing – original draft (equal). **Xiaohan Li:** data curation (equal), formal analysis (equal), software (equal), writing – original draft (equal). **Fang Dong:** data curation (equal), formal analysis (equal), methodology (equal), software (equal), writing – original draft (equal). **Feng Zhang:** conceptualization (lead), formal analysis (equal), funding acquisition (lead), methodology (equal), software (equal), writing – original draft (lead), writing – review and editing (lead).

## Ethics Statement

The authors have nothing to report.

## Consent

The authors have nothing to report.

## Conflicts of Interest

The authors declare no conflicts of interest.

## Supporting information


Data S1.

Data S2.

## Data Availability

This study analyzed publicly accessible datasets, including GSE132903, GSE98793, and GSE1297 datasets from Gene Expression Omnibus (GEO, https://www.ncbi.nlm.nih.gov/geo/query).
