# Supplementary material for: Decoding Alzheimer's Disease With Depression: Molecular Insights and Therapeutic Target
Source: J Cell Mol Med. 2025 Mar 12;29(5):e70454. doi: 10.1111/jcmm.70454 (PMC11903198; doi:10.1111/jcmm.70454)
Supplement: Supplementary file 1 — Data S1. [file JCMM-29-e70454-s001.docx]

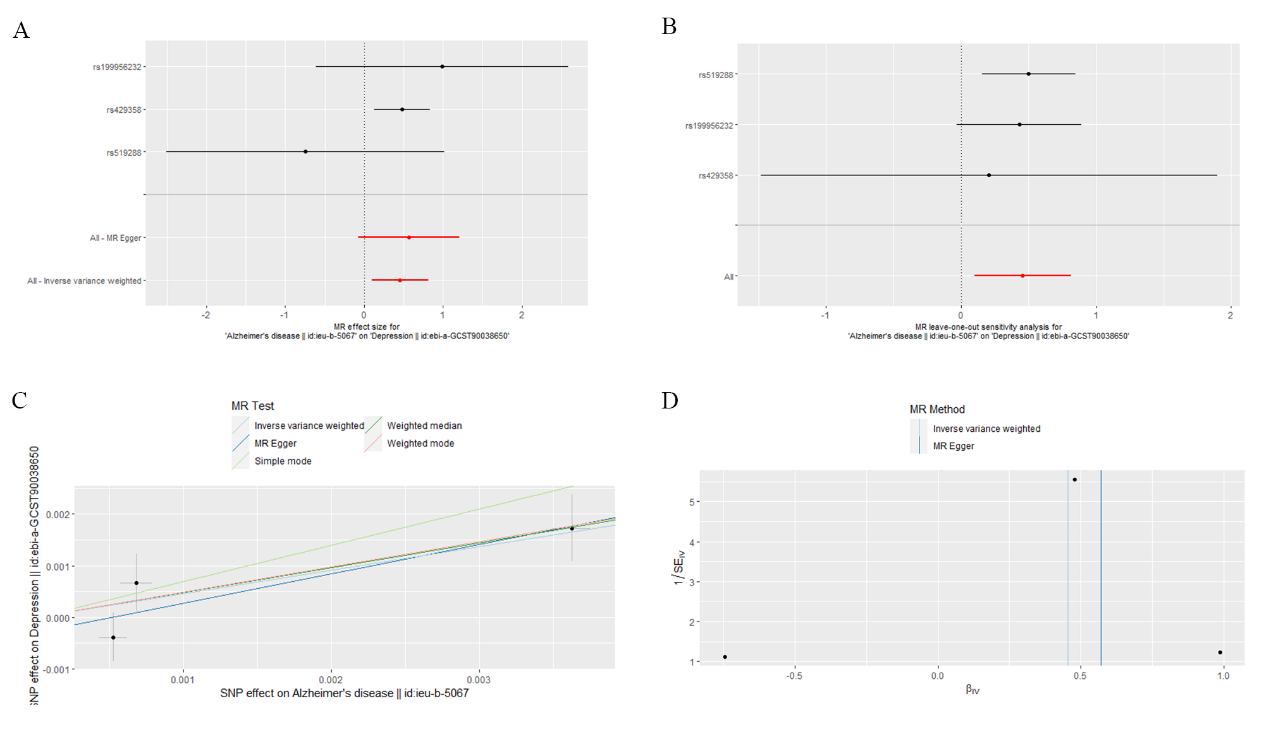


**Fig S1** The causality of AD on depression in Europeans. (A) Forest map, the red dots represent a comprehensive estimate utilizing all SNPs, utilizing the IVW method, the horizontal line represents a 95% confidence interval; (B) MR leave-one-out sensitivity analysis for AD on depression, the black dots indicate that the IVW mean was utilized to evaluate causal influences, excluding a single specific variable in the analysis; (C) Scatter plot, the slope of different colored lines indicated the estimation effect of different MR methods; (D) Funnel diagram, the vertical line denoted the estimated value of all SNPs, and the symmetry of funnel plot showed no obvious horizontal pleiotropy.


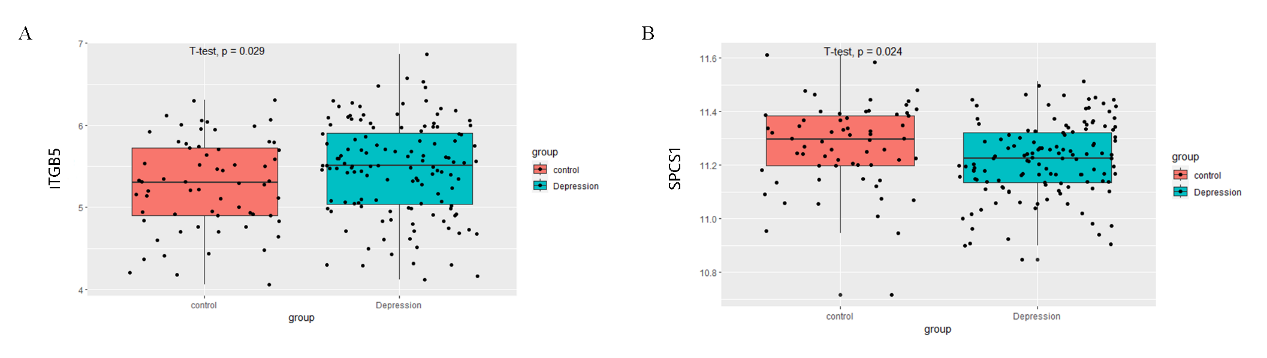


**Fig S2** The levels of two predictive biomarkers in the control and depression groups (GSE98793). (A) The levels of ITGB5 in the control and depression groups; (B) The levels of SPCS1 in the control and depression groups.
